# Facing the Journey Together: Using Digital Media to Support Dementia Care Among Asian Americans

**DOI:** 10.1093/geroni/igaa057.1762

**Published:** 2020-12-16

**Authors:** Angelica Yeh, Marie Mayen-Cho

**Affiliations:** 1 Alzheimer’s Los Angeles, San Gabriel, California, United States; 2 Alzheimer’s Los Angeles, Los Angeles, California, United States

## Abstract

Asians and Pacific Islanders (APIs) in the United States have limited access to dementia care information that is linguistically and culturally appropriate. Alzheimer’s Los Angeles created “Faces of Caregiving”, a video project available with English/Japanese subtitles, documenting in-depth interviews with 7 Japanese/Japanese-American familial care partners of individuals living with dementia. It touched on the personal yet universal aspects of each journey embedded in a particular family context. The 5 video profiles were subsequently shown at 3 community sites to attendees comprised of mostly older-adult APIs. Among 85 attendee responses, approximately 90% stated they were more likely to seek out information on and support for Alzheimer’s disease, felt more open to talking about the disease, and were more likely to advocate and raise awareness for the disease. This program could be replicated for other API communities, allowing individuals to learn more effectively from a peer-to-peer experience in a culturally familiar setting. Part of a symposium sponsored by the Aging Among Asians Interest Group.

